# Investigation of active matrix- metaloproteinase-8 (aMMP-8) as a reference parameter for path control in antimicrobial photothermal therapy (aPTT) using a split-mouth design

**DOI:** 10.1016/j.heliyon.2019.e01661

**Published:** 2019-05-17

**Authors:** J. Deumer, M. Frentzen, M.C. Meinke

**Affiliations:** a MVZ Erstes Zahnärztliches Lasercentrum Berlin, Gatower Straße 296, 14089 Berlin, Germany; b Department of Operative and Preventive Dentistry, Bonn University, Dental Faculty, Germany; c Department of Dermatology, Venereology and Allergology, Charité University Medicine Berlin Campus Charité Mitte, Germany

**Keywords:** Dentistry

## Abstract

**Objectives:**

This retrospective data-collection study aims to explain how the active matrix-metalloproteinase-8-titer (aMMP-titer) influences the immune response of the subject. This is done through monotherapy scaling and root planing (SRP) which is then compared to SRP combined with antimicrobial photothermal therapy (aPTT, Emundo®).

**Methods:**

Data collection was monocentric, randomized and split-mouth based. A study group of twenty patients with chronic periodontal disease with a periodontal pocket depth (PPD) 4 mm ≤ PPD ≤8 mm, a periodontal screening index (PSI: > 3), and a gingival recession ≤2 mm were selected.

A diode laser, manufactured by A.R.C. Laser GmbH, with 810 nm wavelength was used. This device implemented three different light transmission systems for transgingival and intra-gingival irradiation. Power settings between 200 and 300 mW were deployed for 10 s during all treatment steps. The photothermic dye of EmunDo® system (A.R.C. Laser GmbH) was infracyaningreen.

The adjuvant effect of the antimicrobial photothermal therapy (aPTT) with EmunDo® in combination with conventional SRP on the teeth 15 and 35 was compared with the results of monotherapy SRP on teeth 25 and 45.

**Results:**

A reduction of the aMMP-8-titer in gingival crevicular fluid (GCF) was observed in both groups (follow up group and control group) after one month. However; the decrease in the follow up group under SRP in combination with aPTT was significantly more pronounced. The periodontal pocket depths was reduced in both treatment groups. The periodontal probing depth (in mm) shows a larger decrease of the periodontal pocket depth within the follow up group (SPR with aPTT) compared with the control group (SRP).

**Conclusion:**

The aMMP-8-titer showed differences in both groups prior to and after treatment. Active matrix-metalloproteinase-8 (aMMP-8) as a reference parameter for path control in antimicrobial photothermal therapy (aPTT) seems acceptable.

## 1 Introduction

In respect to etiology and pathogenesis, chronic periodontitis is defined as a bacterial disease [1, 2]. The extent of the disease is determined by the patient's immune response, this being based on genetically manifested polymorphisms [3, 4, 5, 6]. This plaque induced periodontitis leads to the release of the matrix-metalloproteinase-8 (MMP-8) [7]. The inactive matrix-metalloproteinase (proMMP) is activated by the ‘cystein switch’ and is the catalyst for collagen type 1 property [3, 4, 5, 6]. This oxidative conformational change depends on a number of factors; bacterial proteinases, temperature, concentration of hydrogen ions (pH-value), calcium (Ca^2 +)^ and oxygen radicals. Therefore, active matrix-metalloproteinase-8 (aMMP-8) could be an indicator of tissue disintegration [8]. The laboratory-chemical detection of aMMP-8 in the gingival crevicular fluid (GCF) enables a differentiation of the inflammatory status and tissue degradation of periodontitis. The clinical parameters (periodontal pocket depth and bleeding on probing) indicate the current state of clinical attachment loss, evidence by culture of aMMP-8 of the sulcus fluid provides information on the current collagen degradation [9, 10, 11, 12, 13, 14]. An increase in the concentration of aMMP-8 in the sulcus fluid of periodontal patients can be detected in the saliva [7,15,16]. Vis-a-vis the bloodstream, mutual interactions can be stimulated which can promote bacterial growth [17]. These increased proteinase activities; besides periodontitis, can also be responsible for arthritis [18, 19, 20, 21] cardiovascular [17, 22, 23, 24, 25] and cancer-related diseases. Activation of the MMP takes place within the scope of the immune defense and can ultimately lead to the decay and destruction of the tooth holding apparatus [26,27]. The prerequisite for a successful treatment of chronic periodontitis is the removal of the concrements at the root surface [28] as well as the elimination of periodontal bacteria [29]. Laser systems can meet both criteria: cleaning as well as providing an antimicrobial effect [30,31]. The advantage of the laser treatment is preventing the formation of resistance [32]. The antimicrobial photothermal therapy (aPTT) is a laser-assisted therapy with a diode laser in combination with infracyaningreen. In this retrospective data survey, the immune response of the subject (aMMP-8) is to be examined and compared to other clinical findings (periodontal pocket depth and bleeding on probing) under monotherapy SRP are compared with SRP in combination with aPTT. A diode laser with a wavelength of 810 nm was used accordance with the manufacturer's instruction (A.R.C. Laser GmbH) with three different light transmission systems and corresponding power settings. The EmunDo® system (also A.R.C. Laser GmbH) with dye infracyaningreen was used as a photothermic component. According to Engelschalk and Kranz et al., the therapeutic effect of the infracyaningreen is mainly in photothermics; with temperature intervals of over 42 °C, which then leads to protein denaturation [10,33,34]. Following local application, the dye immediately binds 98% to plasma proteins, with a preference made to α1-lipoproteins [35]. The dye spreads homogeneously into the tissue for a few seconds [36,37]. The sensitizer is not absorbed into the intestine, thus causing no damage [38,39]. EmunDo® is an iodide-free dye. Ideally it is suited as a photoactive substance because of its amphiphilic property [40,41]. By itself, this dye has no disinfectant properties, and the therapeutic effect is limited solely by the application time with the diode laser. This is achieved through photothermics [10]. The aPTT with the wavelengths of 810 nm has a high penetration depth and can reach depths of up to 5–8 mm [42].

## 2 Methods

For data collection (monocentric, randomized, split-mouth-based) twenty patients with a diagnosis of chronic periodontitis were selected. Teeth with a periodontal probing depth (PPD) of 4 mm ≤ PPD ≤8 mm, periodontal screening index (PSI) of 3 ≤ PSI ≤4 and a gingival recession ≤ 2mm were recorded in the applicant pool. Further findings were not recorded. The patient pool consisted of thirteen female and seven male patients, all aged between 30 and 70 years. The median of the age population is 51 years.

The study was approved by the local Ethics Committee of the Charité - Universitätsmedizin Berlin (EA1/246/18) according to the Declaration of Helsinki (1983).

Informed consent was obtained from all study participants in accordance with ethical and moral standards.

Exclusion criteria of the patient cohort, reactive the last six months;

● anticoagulant medication
● antibiotic use
● nicotine consumption
● blood clotting disorder
● Type-2 diabetes
● vascular disorders
● patients under 18 years of age
● patients with sensitivity to colors and light
● patients with impaired compliance

The first and third tooth quadrant were treated in combination with antimicrobial photothermal therapy (aPTT) and SRP, in the statistics figure we call them `**follow up group**`; second and fourth tooth quadrant were treated only with an SRP, we call them in the statistics `**control group**`. Tooth 15 was the representative of the first tooth quadrant and tooth 25 was the representative of the second tooth quadrant, third 35 and fourth 45 respectively.

The adjuvant effect of the antimicrobial photothermal therapy (aPTT) with Emundo® in combination with SRP in the first and third quadrant was compared to the monotherapy SRP in the second and fourth quadrant. The second premolars present the respective tooth-quadrant.

In addition to active matrix-metalloproteinase-8 (aMMP-8) detection, the periodontal pocket depth (PPD) and the index bleeding on probing (BoP) were used as clinical parameters for the characterization of the periodontal lesion. The survey period was four weeks. The parameters were determined before the trial and four weeks following the treatment.

The sample material for aMMP-8 determination (ng/ml) was measured using a classical ELISA-test (sampling set for aMMP-8: tissue status in periodontitis, laboratory for medical diagnostics was MVZ GbR in Berlin-Potsdam, Germany) for the quantitative aMMP-8 determination the gingival crevicular fluid (GCF) from the above-mentioned tooth pockets was used. The gingival crevicular fluid (GCF) was taken off with litmus paper with remained in the gingival sulcus for thirty seconds. The sample was then packed into sterile shipping tubes. The pool samples (four test strips/tooth) were determined by a classical sandwich ELISA-test in ng/ml at the laboratory for medical diagnostics (Medizinisches Versorgungszentrum MVZ GbR) in Berlin-Potsdam.

According to the manufacturer, the detection limit of the applied ELISA-test is 0.04 ng/ml. The measurement range is 0,125–1,6 ng/ml (conversion rate is then multiplied by 50). Results outside of the detection limit were recorded as ‘< 5’ or ‘>80’. According to the manufacturer, the sample stability of the gingival crevicular fluid (GCF) on the test-strips is four days at room temperature; or up to a week at 4 °C. In preparation for an ELISA-test, test strips are placed individually in 600μl eluent-solvent. After elution, the test strips are removed and the eluent is then analyzed.

After completion of the pretreatment, the SRP was completed within a time-frame of seven days encompassing two sessions. After conventional periodontal treatment with an air scaler (Sonicflex, in combination with Paro-lace-set 60–62), the antimicrobial photothermic therapy was continued within seven days (+/- two days). The aPTT was repeated after one day. According to the manufacturer, three light guides (transgingival handpiece, bulb and bare fiber) were used for light propagation. The aPTT with infracyaningreen included on the first quadrant in the maxilla and the third quadrant in the mandible. In order to prevent a distribution of the sensitizer across the boundary regions of the teeth 11, 21 and 31, 41, a barrier protection made of silicone (known as Liquidam®, Discus Dental GmbH) was applied. The sensitizer was applied just before the start of the treatment and applied along the marginal gingiva in the first tooth quadrant. The aPTT was performed in three steps:

1.Transgingival irradiation of marginal gingiva (transgingival handpiece with 5 mm spot diameter).

The dye was irradiated with the transgingival handpiece along the marginal gingiva without exposure time. The handpiece was guided from gum papillae to gum papillae in a distance of one centimeter from the surface. Radiation was performed from vestibular and palatinal for 10 s. The entire marginal gingiva was treated in the first quadrant. Finally, the collected saliva and the remaining sensitizer were aspirated in the mouth, laser parameters:

● diode laser, 810 nm
● mode of operation: continuous wave
● output: 300mW
● radiation time: 10 s
● distance from surface: 8–10 mm.

2.Intragingival irradiation with the light guide, called bulb fiber (300 μm fiber with rounded fiber-end).

Dye was once again applied in the first tooth quadrant. The dye was blasted intragingival with the bulb fiber. For this purpose, the fiber was inserted as far as the pocket fundus and led to a marginal gingival margin with flowing, meandering movements. The treatment time was as described above. Finally, the saliva and the residual dyes were suctioned intra-orally, laser parameters:

● diode laser, 810 nm
● mode of operation: continuous wave
● output: 200 mW
● radiation time: 10 s
● contact: intragingival

3.Intragingival irradiation with an optical fiber, named bare fiber (300 μm fiber with planar fiber end).

A repeated application of infracyangreen in the first quadrant took place. The bare fiber was introduced to the pocket floor. The fiber guidance corresponded to that of the bulb fiber. The irradiation was also performed vestibular and palatinal. The parameters corresponded to the settings on the bulb fiber, laser parameters:

● diode laser, 810 nm
● mode of operation: continuous wave
● output: 200 mW
● radiation time: 10 s
● contact: intragingival

This treatment sequence was carried out in third tooth quadrant with all three light guides. Repeat therapy was performed after two days (+/- one day) as previously described. The second and fourth tooth quadrants remained photothermally untreated. After four weeks (+/- three days), the indices (PSI, BoP, Plaque-index after Quigley-Hein, periodontal status, aMMP-8-titer) were again taken to examine treatment results. All materials are listed below:

● Diode laser, 810 nm (manufacturer by A.R.C. Laster GmbH)
● three light guides
  ○ transgingival handpiece with 5 mm width
  ○ bulbfaser (Faser 300 μm with rounded edges)
  ○ barefaser (Faser 200 μm with flat edges)
● photosensitizer, infracyangreen, EmunDo®
● physiological saline solution
● protective glasses
● Liquidam® (from Discus Dental GbmH)

### 2.1 Statistical analyses

All data was analyzed using Excel from Microsoft Office (Home and Student 2013). Statistical evaluation of periodontal pocket depth and enzyme levels (aMMP-8) was performed using the IBM SPSS (Statistical Package for the Social Sciences) statistical software, SPSS Statistics Base and Origin® 2017 Graphing and Analyse for the boxplots. The following test procedures were performed: The data was tested using the Shapiro-Wilk and Wilcoxon test. Because the data was non-parametric and paired, Wilcoxon test was applied for data analysis between the study and the control groups. The level of significance was assumed at p ≤ 0.05 (high significance: p ≤ 0.01, highest significance: p ≤ 0.001).

## 3 Results

In the evaluation of the follow-up, the date from a total of twenty patients were considered in a split-mouth design. There were thirteen female (65%) and seven (35%) male patients. The age distribution of the patients were 40% in the age group 41–50 years, 15% in the age group 61–70 years and 5% in the age group 30–40 years.

### 3.1 Active matrix-metalloproteinase-8 (aMMP-8) courses in gingival crevicular fluid (GCF)

**Fig. 1:**
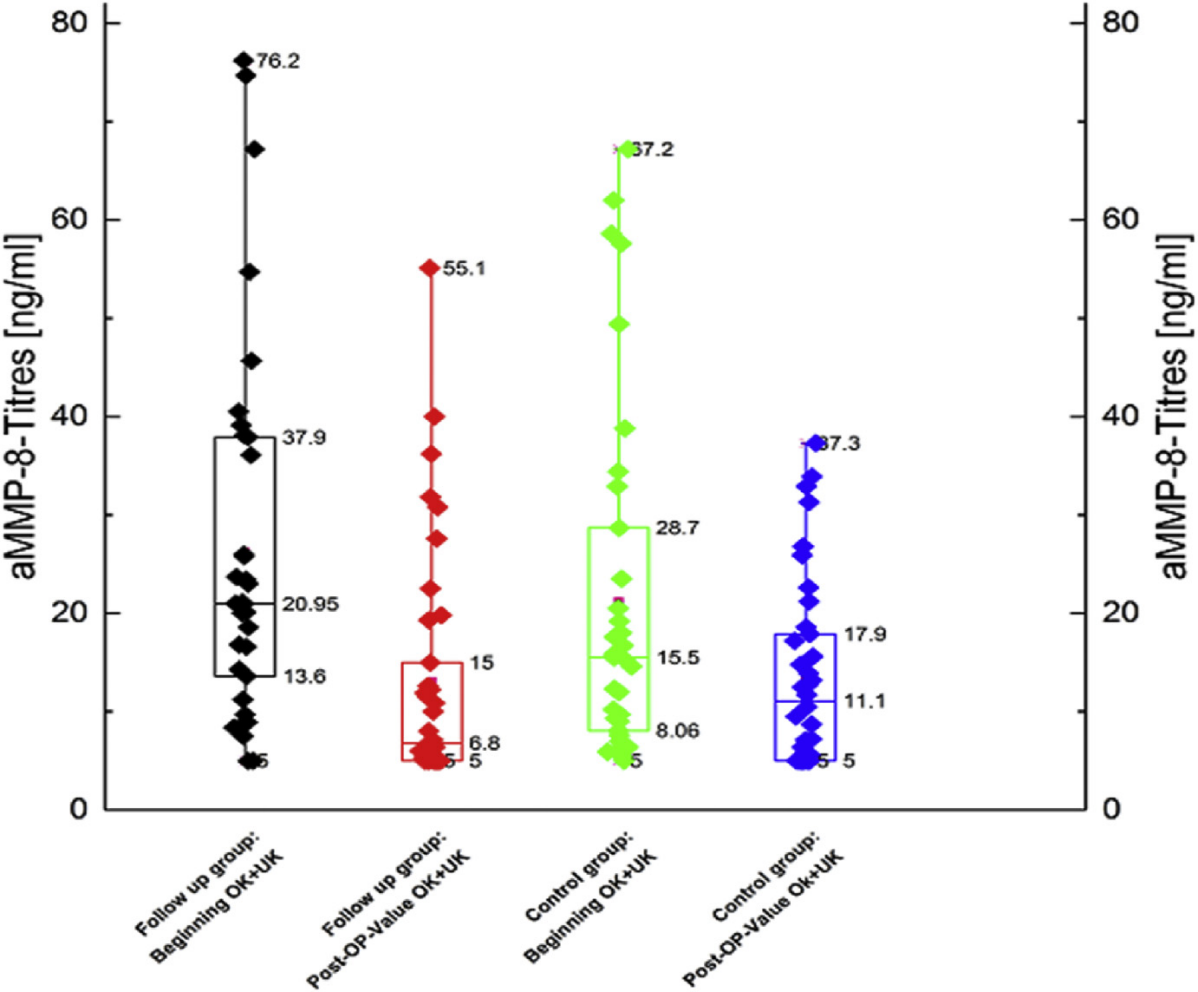
Boxplot of aMMP-8-titer before and after intervention.

The measured aMMP-8-titer (1 ng/ml) shows a larger decrease of the aMMP-8-titer within the study group (addition laser treatment) regarding the unweighted median. This was compared to the control group (no addition laser treatment). The value of the decrease of the aMMP-8-titer is 14.15 ng/ml within the study group. In the control group the decrease was measured at 4.4 ng/ml. An additional view of the weighted mean confirmed a clear decrease in the aMMP-8-titer within the study group compared to the control group. The lowering within the follow up group is > 13 ng/ml, whereas it is < 8 ng/ml within the control group (Fig. 1).

The boxplot of the measured aMMP-8-titer (Unit: 1 ng/ml) shows a larger decrease of the aMMP-8-titer within the follow up group (additional laser treatment) regarding the (unweighted) median, compared to the control group (no additional laser treatment). The value of the decrease of the aMMP-8-titer is 14,15 ng/ml within the follow up group, whereas the decrease is 4,4 ng/ml within the control group. An additional view onto each (weighted) mean value confirms a clear decrease of the aMMP-8-titer within the follow up group compared to the control group**.** The lowering within the follow up group is > 13 ng/ml, whereas it is < 8 ng/ml within the control group. Follow-up group (SRP and aPTT treated) includes the measured values in the first and third quadrant of teeth. The values were measured as a pool decrease on the second premolar (tooth 15 and tooth 35).Control group (SRP treated) includes the measured values in the second and fourth quadrant. The values were also measured as a pool decrease on the second premolar (tooth 25 and tooth 45).

### 3.2 Periodontal pocket depths (PPD)

**Fig. 2:**
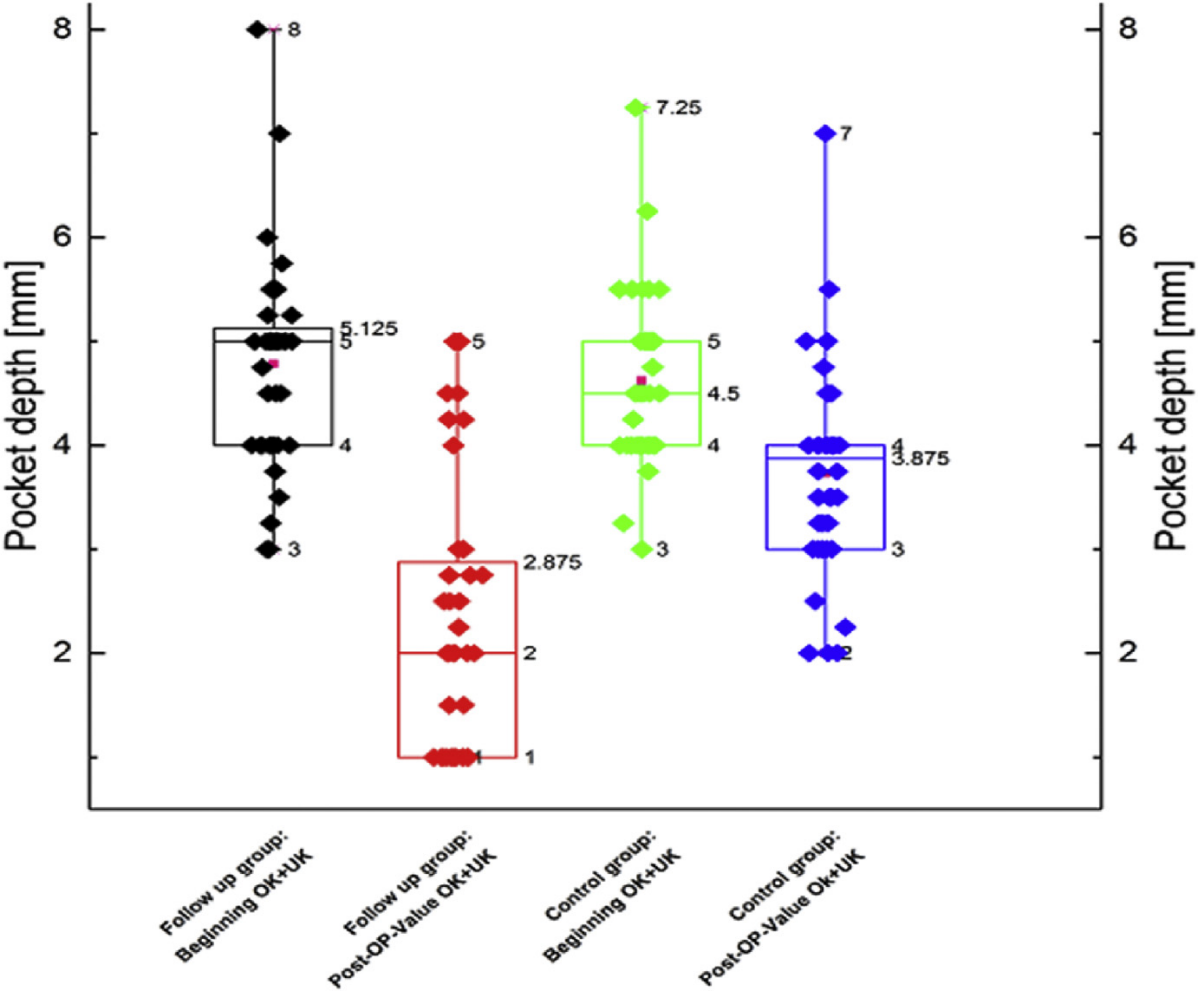
Boxplot of periodontal pocket depth before and after intervention for follow up and control group.

The second graph of the measured periodontal probing depth (in mm) shows a larger decrease of the periodontal pocket depth within the study group (SPR with aPTT) compared with the control group (SRP). The decrease was 3 mm within the study group and 0.7 mm in the control group. In regards to the mean value, the decrease in the periodontal pocket depth of the study group was 2.55 mm in comparison to 0.9 within the control group (Fig. 2).

The second boxplot of the measured periodontal pocket depth (Unit: 1mm) shows a larger decrease of the periodontal pocket depth within the follow up group regarding the median compared to the control group. The decrease is 3mm within the follow up group, and <0.7mm within the control group. Regarding the mean value it is shown that the drop of the periodontal pocket depth within the follow up group is 2,55mm, whereas it is < 0,9mm within the control group. Follow-up group (SRP and aPTT) includes the measured values in the first and third quadrant of teeth. The values were measured mesially and distally on the second premolar (tooth 15 and tooth 35).Control group (SRP treated) includes the measured values in the second and fourth quadrilateral. The values were measured mesially and distally on the second premolar (tooth 25 and tooth 45).

### 3.3 Bleeding on probing (BoP)

The study group and the control group showed identical BoP values at the baseline examination on the upper and lower jaw. In both groups, the BoP had decreased before treatment. The BoP was still negative in all patients of the test group (SRP and aPPT) in the upper and lower jaws even after one month. In the control group (SRP) the BoP had reduced in the upper and lower jaws, thus, 17 out of 20 patients had shown a negative BoP.

### 3.4 Active matrix-metalloproteinase-8 (aMMP-8) and periodontal pocket depth (PPD)

The aMMP-8-titer and periodontal pocket depths decreased in both groups. However; the extent of the decrease in the study group was higher than in the control group (Figs. 1 and 2). There was no correlation between probing depth and aMMP-8-titers in the test and control groups, this was evident both before and after treatment.

## 4 Discussion

### 4.1 Active matrix-metalloproteinase-8 (aMMP-8)

Among other proteinases, the active matrix metalloproteinase-8 (aMMP-8) is one of the main causes of periodontal tissue degradation [13,43,44]. A significant effect of treatment with antimicrobial photothermal therapy (aPTT) with Emundo® was compared to treatment solely with SRP. The effects of these treatments were then examined one month following the initial study. Treatment with aPTT and SRP yielded more pronounced positive results than treatment only with SRP. It is important that an SRP be administered first, that way the blood index of the periodontitis is reduced. Otherwise, the light of the laser will be absorbed into the blood and not in the dye. The study from Günther failed to make this connection. In his study, the process was done in a different order. This yielded, through aPDT, no improved success. He also stated that aPDT was useless. Although not directly comparable with other similar design, comparable results were also yielded, assuming SRP was performed before antimicrobial photodynamic therapy (aPDT) [10,29,32]. In the Günter study, such effective treatment results were not yielded, because he treated first with aPDT and then with SRP This is due to insufficient pretreatment and because the hemoglobin laser power and the use of a different dye; which had a different healing result [45]. The presence of deposits on the teeth (plaque, tartar or concrements) which block light propagation do not allow the dye to have the desired healing effect [46,47]. Indocyaningreen works through photothermics [10]. The SRP must be performed before aPTT because of light propagation. Blood must be reduced in order to promote dye absorption [32]. The MMP-8 is a zinc-dependent endopeptidases. The collagenase is secreted as inactive proenzymes and require enzymatic cleavage of the propeptide domain for activation. This process (cystein-switch) is very heat sensitive [48, 49, 50]. However; the inhibitors of aMMP-8 (tissue inhibitor of matrix-metalloproteinase: TIMPS) are resistant to heat [51,52]. The aPTT temperature increase from 45 °C to 65 °C and is sufficient for destruction of the collagenase but not the TIMPS [10]. The MMPs are zinc-dependant endopeptidases. They are secreted as inactive proenzymes and require enzymatic cleavage of the propeptide domain for activation. This process, cystein-switch, is very heat sensitive [48, 49, 50]. However; the inhibitors of aMMP-8 (tissue inhibitor of matrix-metalloproteinase: TIMPS) are heat-resistant [51,52]. This could be the cause of the decrease of the aMMP-8.

### 4.2 Periodontal pocket depth

The aMMP-8-titer is conform with the degree of periodontal tissue degradation. This is why the periodontal pocket depth was taken as a measure of periodontal destruction [7,43,53, 54, 55]. On average, the decrease in probing depths in the test group were significantly higher (2.55 mm) in comparison to the control group (1.005 mm) These probing depth reductions in the test group were no expected after one month as tissue-remodeling usually takes six months [56, 57, 58]. An explanation could be that the thermal effects caused by infracyaningreen induced an alteration in the collagenous fiber. This effect can also be observed in the shrinkage of the uvula in anti-snoring therapy or in anti-wrinkle therapy. The inner ‘shrinking’ of collagen fiber has been a central component in laser and radiofrequency therapy in plastic surgery for years. The temperature increase from 45 °C to 65 °C is sufficient for destruction of the collagen, causing the tissue to shrink up to 30% [59,60]. The collagen destruction is then followed by collagen regeneration. The reduction of the periodontal pocket depths in comparison to test and study groups were clearly visible (Figs. 1 and 2). This would mean that aPTT with Emundo® was successful due to the principle of collagen tightening. A diode laser alone could not yield these results, as the wavelength has too high of a penetration depth. In addition, the amount of energy applied in aPTT was too low to cause collagen destruction. However; in combination with the dye, temperatures between 45 C and 65 C were yielded [10,61,62]. This increase in temperature is enough to destroy the collagen and reduce the periodontal pocket depth, elimination the recession where the bacteria could hide. Compared with the control group, the addition of aPTT in the study group proves the additive therapeutic effect. Compared to the processes where dyes toluidine blue and methylene blue were added, infracyaningreen yielded much better results. This can be justified by the thermal power of the diode laser. In contrast to SRP alone, tissue disinfection is improved. In the case of antimicrobial photodynamic therapy (aPDT) with toluidine blue or methylene blue, lipopolysaccharides (LPS) remain as a product of the bacterial membrane. LPS is also extremely cytotoxic in nanomolecular concentrations [25,63] and prevents the formation of an attached gingiva [15,64, 65, 66]. The aPDT with methylene blue and toluidine blue led to a more moderate increase in the inflammatory mediators and thus to an increase in the aMMP-8 concentration [29,67]. The immunological reactions against thermally altered bacteria or their residues in remaining pockets under the aPTT remain unknown. The thermal, infra-reding-mediated effect may lead to a modulation of the wound surface caused by SRP. This process resembles the coagulation of a diffuse bleeding would in general surgery. The aPTT is in the field of lower-level laser therapy. Laser-activated natrium-kalium-ATPase **(**Na^+^/K^+^)-ATPase) activates the membrane and the membrane potential is changed. The calcium-ATPase (Ca^2 +^ -ATPase) is increased intracellularly, and calcium (Ca^2+^)-concentration increases. This leads to an increased mitotic rate and cell proliferation. The regenerative processes of the gingiva were accelerated parallel to the removal of inflammation [68, 69, 70, 71].

### 4.3 Active matrix-metalloproteinase-8 (aMMP-8) and periodontal pocket depths (PPD)

Periodontal pocket depths were reduced in both treatment groups (Fig. 2). Although the aMMP-8 titer is consistent with the degree of periodontal tissue degradation [7], there is no correlation between aMMP-8 and probing depth (correlation coefficient). A correlation to the established probing depth could not be determined (correlation coefficient). The lack of a correlation should be due to time-delayed patterns of repair or regeneration processes of the pockets and **their immunological activity**. In 2009, Marcaccini et al.and in 2011 Sorsa et al., described an increase in the aMMP-8 values in the blood plasma in periodontal patients and a correlation of the proteinase elevation and its sequelae diseases. They speculated the presence of a pro-inflammatory condition in the body [25,54]. This relationship between periodontal disease and general disorders has been confirmed in any further studies [72,73]. However; no reversal in the interaction between general diseases with an increase of usual inflammatory parameters and periodontitis has been demonstrated.

## 5 Conclusion

The active matrix-metalloproteinase-8 (aMMP-8) represents the differences before and after periodontal treatment and is therefore suitable as a reference marker. The antimicrobial photothermal therapy (aPTT) with Emundo® was useful as an adjuvant measure following a SPR, recognizable by significant to highly significant parameters (aMMP-8, PPD, BoP) compared to SRP alone and is therefore a promising treatment.

Histological investigations are needed in order to evaluate the modulating effects on the wound surface, in the periodontal pocket, as well as the ‘inside shrinking’ in the periodontal tissue following aPTT. The aPTT with Emundo® should be optimized under controlled conditions. In this follow-up, the aPTT was performed with infracyaningreen following two profession teeth cleaning procedures. There was a noted reduction in BoP immediately following the SRP. The aPTT was repeated after one to two days.

## Declarations

### Author contribution statement

Jeannette Deumer: Conceived and designed the experiments; Performed the experiments; Analyzed and interpreted the data; Contributed reagents, materials, analysis tools or data; Wrote the paper.

Matthias Frentzen: Conceived and designed the experiments; Analyzed and interpreted the data.

M. C. Meinke: Analyzed and interpreted the data.

### Funding statement

This research did not receive any specific grant from funding agencies in the public, commercial, or not-for-profit sectors.

### Competing interest statement

The authors declare no conflict of interest.

### Additional information

No additional information is available for this paper.
